# Unemployment rate, opioids misuse and other substance abuse: quasi-experimental evidence from treatment admissions data

**DOI:** 10.1186/s12888-020-02981-7

**Published:** 2021-01-10

**Authors:** Sunday Azagba, Lingpeng Shan, Fares Qeadan, Mark Wolfson

**Affiliations:** 1 Department of Family and Preventive Medicine, University of Utah School of Medicine, Salt Lake City, UT 84108 USA; 2 Department of Social Medicine, Population and Public Health, University of California Riverside School of Medicine, Riverside, CA 92501 USA

**Keywords:** Substance abuse treatment, Treatment admissions, Opioids, Economic conditions; unemployment rate

## Abstract

**Background:**

The relationship between economic conditions and substance abuse is unclear, with few studies reporting drug-specific substance abuse. The present study examined the association between economic conditions and drug-specific substance abuse admissions.

**Methods:**

State annual administrative data were drawn from the 1993–2016 Treatment Episode Data Set. The outcome variable was state-level aggregate number of treatment admissions for six categories of primary substance abuse (alcohol, marijuana/hashish, opiates, cocaine, stimulants, and other drugs). Additionally, we used a broader outcome for the number of treatment admissions, including primary, secondary, and tertiary diagnoses. We used a quasi-experimental approach -difference-in-difference model- to estimate the association between changes in economic conditions and substance abuse treatment admissions, adjusting for state characteristics. In addition, we performed two additional analyses to investigate (1) whether economic conditions have an asymmetric effect on the number of substance use admissions during economic downturns and upturns, and (2) the moderation effects of economic recessions (2001, 2008–09) on the relationship between economic conditions and substance use treatment.

**Results:**

The baseline model showed that unemployment rate was significantly associated with substance abuse treatment admissions. A unit increase in state unemployment rate was associated with a 9% increase in treatment admissions for opiates (β = 0.087, *p* < .001). Similar results were found for other substance abuse treatment admissions (cocaine (β = 0.081, *p* < .001), alcohol (β = 0.050, *p* < .001), marijuana (β = 0.036, *p* < .01), and other drugs (β = 0.095, *p <* .001). Unemployment rate was negatively associated with treatment admissions for stimulants (β = − 0.081, *p* < .001). The relationship between unemployment rate and opioids treatment admissions was not statistically significant in models that adjusted for state fixed effects and allowed for a state- unique time trend. We found that the association between state unemployment rates and annual substance abuse admissions has the same direction during economic downturns and upturns. During the economic recession, the negative association between unemployment rate and treatment admissions for stimulants was weakened.

**Conclusion:**

These findings suggest that economic hardship may have increased substance abuse. Treatment for substance use of certain drugs and alcohol should remain a priority even during economic downturns.

## Background

Substance abuse, the harmful or hazardous use of psychoactive substances, remains a significant public health problem. In 2017, approximately 74,000 persons died of drug-related causes, and about 36,000 died of alcohol-related causes (excluding accidents, homicides, and prenatal exposure) [1]. The United States has experienced an increase in the prevalence of illicit drug use, from 8.3% in 2002 to 11.2% in 2017 [2, 3]. There has also been a recent rise in the use of marijuana from 14.5 million (5.8%) in 2007 to almost 41 million (15%) in 2017 [2, 3]. In contrast, alcohol dependence decreased from 18.1 million (7.7%) in 2002 to 17.3 million (6.6%) in 2013 [2]. In 2017, almost 17 million persons (6.1%) reported heavy alcohol use in the past month, and nearly 67 million (24.5%) reported binge alcohol use, a slight increase from 2016 [2].

The relationship between economic conditions and substance abuse is ambiguous. Several studies have found that tighter budgets during economic crises impacted drinking behaviors, including less alcohol consumption, with people switching to cheaper products and drinking at home rather than drinking at bars [4–7]. However, prior studies have documented that economic downturns, along with their related stresses such as job loss, are associated with increased problematic drinking [8, 9]. There is also evidence that unemployment is strongly associated with problematic substances use, including the use of alcohol, marijuana, and illicit drugs [10]. An international study from Australia found that economic downturns were significantly associated with the frequency of marijuana use among young adults, and a procyclical relationship was found for the frequency of drinking [5]. Furthermore, multiple studies have found that sales and use of marijuana and other illicit drugs increased among young adults and teenagers as the unemployment rate decreased [11, 12]. A study conducted among African American adults in Milwaukee’s inner city found that those employed full-time tested positive for cocaine less often than those who were unemployed, and those employed part-time had higher rates of testing positive than those employed full-time [13].

Substance abuse treatment admissions can be used as an indicator of excessive or problematic substance use. However, due to a lack of available quality drug abuse and treatment data, existing research has been limited to investigating general substance use treatment admissions (not drug-specific) or restricted to certain subpopulations. In addition, few studies have examined the association between national economic conditions and substance use [5, 11, 12, 14].

A recent study in the U.S. based on self-reported data found that economic downturns led to increases in the intensity of prescription pain reliever use as well as opioid substance use disorders, especially among working-age white males with low education [15]. The present study expands on prior work, examining the relationship between economic conditions and substance use, using admissions data from the 1993 to 2016 Treatment Episode Data Sets (TEDS) and a more diverse set of illicit drug categories (i.e., alcohol, marijuana, opioids, stimulants, cocaine) [16].

## Methods

### Data

TEDS is a national admission-based data system administrated by the Center for Behavioral Health Statistics and Quality of the Substance Abuse and Mental Health Services Administration (SAMHSA). Since 1992, the TEDS system has compiled data from each state to track annual discharges and admissions to public and private substance abuse facilities that receive public funding. Treatment facilities that receive public funds or are licensed or certified by state substance abuse agencies are required to report data on all clients, regardless of health insurance status. The TEDS system comprises two major components: the Admissions Data Set and the Linked Discharge Data Set. Both data sets provide demographic, clinical, substance service characteristics and settings, employment status, presence of psychiatric problems, prior history, route of administration, insurance status, and source of payment for all patients 12 years of age and older. In addition, three (primary, secondary, and tertiary) substances of abuse, their route of administration, frequency of use, age at first use, and a source of referral to treatment are recorded for each admission. TEDS classifies substance use into seven categories: alcohol, marijuana/hashish, opiates (heroin, non-prescription methadone, and other opiates and synthetics), cocaine, stimulants (methamphetamine, other amphetamines, and other stimulants), other drugs, and none reported. We used the TEDS admissions data set from 1993 to 2016, excluding the “none reported” category.

### Measurement

#### Dependent variables

State identifiers in TEDS were used to derive the state-level aggregate number of treatment admissions for six categories of primary substance use (alcohol, marjuana/hashish, opiates, cocaine, stimulants and other drugs) for each year individually. Additionally, we used a broader outcome for the state-level aggregated number of treatment admissions, including primary, secondary, and tertiary diagnosis for any each category of substance use.

#### Independent variables

Our main dependent variable was economic condition. We used state unemployment rates to represent the economic condition. State unemployment rates and median household income (in thousand dollars) for each state were obtained from the Bureau of Labor Statistics [17–19]. We also obtained state laws on medical marijuana laws fromProCon.org [20], state alcohol taxes from the tax policy center [21], and the insurance coverage rate for each state from the U.S. Census Bureau [22]. We followed the Census Bureau’s recommendations for obtaining insurance coverage rates and used the Health Insurance Historical Tables - Original Series for 1993 through 1998, the Current Population Survey Annual Social and Economic Supplement (CPS ASEC) data to estimate 1999 through 2007, and the American Community Survey (ACS) for rates after 2007. These two estimates differ slightly but parallel in change between 2009 and 2012. Additional state-level characteristics including the log of population, mean age, percentage of the state population that is male, and percentage of the population that is white were calculated using U.S. Population Data through the National Cancer Institute (NCI) [23]. The inflation-adjusted beer excise tax was measured in each state at the 2018 price level.

We created a dichotomous indicator, economic trend, to test whether economic conditions had an asymmetric effect on substance use treatment admissions in economic upturns and downturns. When unemployment was higher than in the previous period, the economy experienced a downward trend. Otherwise, the economy was under expansion. We additionally created a recession indicator. The two periods, 2001 and 2008–2009, were considered recessions with negative economic growth, in accordance with the National Bureau of Economic Research, Inc assessment [24].

### Statistical analysis

We used difference-in-difference (DID) models to estimate whether changes in economic conditions were associated with changes in substance use treatment admission. Generally, DID is a quasi-experimental design used to estimate the effect of a specific policy or intervention (state unemployment rate in our study) by comparing the changes in outcomes over time between the treatment group and control group [25]. The outcome variable, the state-level aggregate number of treatment admissions, was log-transformed in order to address potential skewness. Multivariable linear regressions models were used to assess the association between the economic condition (state unemployment rate) and substance use treatment admissions. Model 1 adjusted all listed state-level characteristics, including log of population, mean age, percentage of state population that is male, percentage of state population that is white, state insurance coverage rate, state median household income (in thousands), medical marijuana laws, and survey year. Model 1 additionally adjusted for census division fixed effects to capture unobserved confounders that cluster among neighboring states at the division level. State beer taxes were adjusted for alcohol substance use treatment only (see eq. (1)).

1 $$ {Y}_{st}\sim {\beta}_1\ast Eco{n}_s+{\beta}_2\ast {Year}_t+{\beta}_3\ast {Division}_s+\sum {\beta}_j{X}_{st}+{\epsilon}_{st} $$

Model 2 adjusted for state fixed effects to control unobserved confounding influences that are time-invariant and state-specific. We used variance inflation factors (VIF) to check for multicollinearity in covariance between the included state-level characteristics and state fixed effects. We elected not to include the log of population and percentage of the state population that is white in Model 2 (VIF > 10) (see eq. (2)).

2 $$ {Y}_{st}\sim {\beta}_1\ast Eco{n}_s+{\beta}_2\ast {Year}_t+{\beta}_3\ast {State}_s+\sum {\beta}_j{X}_{st}+{\epsilon}_{st} $$

Model 3 added interaction between state and year to Model 2, in order to allow for a state-unique time trend and control for unobserved state-level factors that evolve at a constant smooth function (see eq. (3)). Models 1–3 were repeated for the broader substance use treatment variable.

3 $$ {Y}_{st}\sim {\beta}_1\ast Eco{n}_s+{\beta}_2\ast {Year}_t+{\beta}_3\ast {State}_s+{\beta}_4\ast {State}_s\ast {Year}_t+\sum {\beta}_j{X}_{st}+{\epsilon}_{st} $$

To investigate whether economic conditions have an asymmetric effect on the number of substance use treatment admissions during economic upturns and downturns, following the work by Mocan and Bali [26], Model 4 modified Model 3 by defining the number of substance use admissions as an asymmetric function of two decomposed unemployment rates during economic downturns (state unemployment rate in periods when it is higher than the prior period) and economic upturns (state unemployment rate in periods when it is lower than the prior period) (see eq. (4)).

4 $$ {Y}_{st}\sim {\beta}_1\ast Eco{n}_{down}+{\beta}_2\ast Eco{n}_{up}+{\beta}_3\ast {Year}_t+{\beta}_4\ast {State}_s+{\beta}_5\ast {State}_s\ast {Year}_t+\sum {\beta}_j{X}_{st}+{\epsilon}_{st} $$

Econ_down_ and Econ_up_ were constructed using state unemployment rates. Econ_down_ equals unemployment rate in periods when it is higher than the prior period, and Econ_up_ equals 0 during economic downturns. Econ_up_ equals unemployment rate when it is lower than the prior period, and Econ_down_ equals 0 during economic upturns. The **X**st is a vector of other covariates included in the model.

5 $$ {Y}_{st}\sim {\beta}_1\ast {R}_{st}+{\beta}_2\ast {UR}_{st}+{\beta}_3\ast {R}_{st}\ast {UR}_{st}+{\beta}_3\ast {Year}_t+{\beta}_4\ast {State}_s+{\beta}_5\ast {State}_s\ast {Year}_t+\sum {\beta}_j{X}_{st}+{\epsilon}_{st} $$

To test the moderation effects of economic recessions (2001, 2008–09) on the relationship between economic conditions and substance use treatment, Model 5 modified Model 3 by adding the economic recession indicator (R) and an interaction term between the state unemployment rate (UR) and economic recession indicator (R) (see eq. (5)). All tests were two-sided and used a 5% significance level. All of the analyses were performed using SAS 9.4 (SAS Institute, Inc., Cary, NC) and R 3.5 (R Foundation for Statistical Computing, Vienna, Austria).

## Results

### Economic condition

**Fig. 1:**
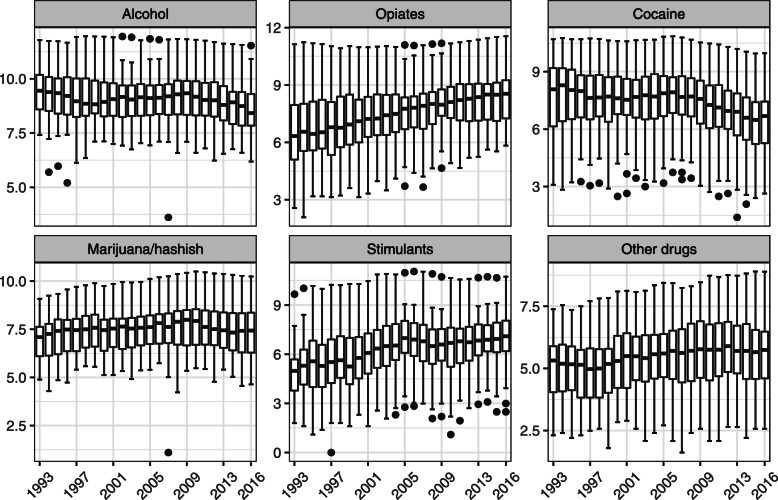
Distribution of admissions for those aged 18 years old and older by primary substance use from 1993 to 2016. The number of substance use treatment admissions was presented after log transformation

Figure 1 presents substance use treatment admissions from 1993 to 2016. The median number of state substance use treatment admissions for opiates and stimulants increased while the admissions for alcohol and cocaine decreased during the study period (Fig. 1).

Table 1 presents the association between the state unemployment rate and annual admissions to substance abuse treatment facilities. Unemployment was significantly associated with substance abuse treatment. In Model 1, a unit increase in unemployment rate was associated with a 9% [(exp (0.087)-1)*100%] increase in opioid treatment admissions ($\hat{\beta}$ =0.087, *p* < .001). Similar associations were found in treatment admissions for cocaine ($\hat{\beta}$ =0.081, *p* < .001), alcohol ($\hat{\beta}$ =0.050, *p <* .001), marijuana ($\hat{\beta}$ =0.036, *p* < .01), and other drugs ($\hat{\beta}$ =0.095, *p <* .001). However, states with higher unemployment rates had a lower number of treatment admissions for stimulants ($\hat{\beta}$ = − 0.081, *p <* .001). In Model 2, which adjusted for state fixed effects instead of division effects as in the first model, a similar association was found between state unemployment rate and substance use treatment admissions, except the association was not significant for opiates admissions ($\hat{\beta}$ =0.002, *p* = 0.84). Similarly, in Model 3, after adjusting for state-year interaction in addition to Model 2 covariates, unemployment rate was positively associated with alcohol admissions ($\hat{\beta}$ =0.026, *p* < .001) and admissions for other drugs ($\hat{\beta}$ =0.049, *p <* .001), but negatively associated with stimulants admissions ($\hat{\beta}$ = − 0.067, *p <* .001). State median household income and population were significantly associated with all substance abuse treatment admissions. Similar results were found for the association between unemployment rate and broader estimated annual admissions.

**Table 1:** Association between unemployment rate and substance abuse treatment admissions, 1993–2016

Primary diagnosis	Model 1			Model 2			Model 3		
	$\hat{\beta}$	SE($\hat{\beta}$)	*p*-value	$\hat{\beta}$	SE($\hat{\beta}$)	*p-*value	$\hat{\beta}$	SE($\hat{\beta}$)	*p-*value
Opiates	0.087	0.012	**<.001**	0.002	0.010	0.84	0.005	0.008	0.54
Cocaine	0.081	0.014	**<.001**	0.033	0.011	**<.01**	−0.004	0.009	0.64
Alcohol	0.050	0.011	**<.001**	0.039	0.007	**<.001**	0.026	0.007	**<.001**
Marijuana/hashish	0.036	0.012	**<.01**	0.024	0.009	**<.01**	0.006	0.009	0.49
Stimulants	−0.081	0.016	**<.001**	−0.057	0.012	**<.001**	− 0.067	0.010	**<.001**
Other drugs	0.095	0.013	**<.001**	0.068	0.011	**<.001**	0.049	0.010	**<.001**
Any diagnosis									
Opiates	0.080	0.014	**<.001**	0.009	0.010	0.34	0.010	0.008	0.22
Cocaine	0.063	0.012	**<.001**	0.030	0.009	**<.001**	−0.002	0.008	0.80
Alcohol	0.041	0.011	**<.001**	0.029	0.007	**<.001**	0.017	0.007	**0.01**
Marijuana/hashish	0.029	0.011	**<.01**	0.025	0.008	**<.01**	0.010	0.007	0.16
Stimulants	−0.066	0.014	**<.001**	−0.033	0.010	**<.01**	−0.040	0.009	**<.001**
Other drugs	0.098	0.014	**<.001**	0.065	0.011	**<.001**	0.050	0.010	**<.001**

*p* < 0.05 is presented in bold. Primary diagnosis represents primary substances cited for alcohol or drug treatment. Any diagnosis represents that the substance was one of the primary, secondary, or tertiary substances cited for alcohol or drug treatment

Model 1 adjusted all listed state-level characteristics, including log of population, mean age, percentage of state population that is male, percentage of state population that is white, state insurance coverage rate, state median household income (in thousands), medical marijuana laws, census division fixed effects and survey year. State beer taxes were adjusted for alcohol substance use treatment

Model 2 adjusted for state fixed effects to control unobserved confounding influences that are time-invariant and state-specific in addition to Model 1 state-level characteristics

Model 3 added interaction between state and year to Model 2, in order to allow for a state- unique time trend and control for unobserved state-level factors that evolve at a constant smooth function

### Unemployment symmetric effects and economic recession

Table 2 shows the results of the association between state unemployment rates and annual admissions to substance abuse treatment facilities in economic downward and upward trends (Model 4). Compared to the original Model 3 results, unemployment rates remain negatively associated with annual admissions for stimulant treatment during economic downturns ($\hat{\beta}$ = − 0.070, *p <* .001) and economic upturns ($\hat{\beta}$ = − 0.092, *p* < .001). Likewise, unemployment rates were positively associated with annual alcohol and other drug treatment admissions during economic downturns and upturns. The state unemployment rates were negatively associated with annual cocaine treatment admissions during economic downturns and economic upturns ($\hat{\beta}$ = − 0.037, *p <* .001), but the association was not significant during economic downturns ($\hat{\beta}$ = − 0.009, *p* = 0.35). We found that the association between state unemployment rates and annual substance abuse admissions has the same direction during economic downturns and upturns. Therefore, unemployment rate appears to have a symmetric effect given that Econ_up_ and Econ_down_ coefficients were not statistically significantly different (except for marijuana/hashish, stimulants, cocaine).

**Table 2:** Association between state unemployment rate and annual admissions to substance abuse treatment facilities in economic downward and upward trends

Mode 4		Baseline model: state unemployment rate	State unemployment rate in periods when it is higher than the prior period	State unemployment rate in periods when it is lower than the prior period
Opiates	$\hat{\beta}$	0.005	0.004	−0.001
	SE($\hat{\beta}$)	0.008	0.008	0.010
	*p-*value	0.54	0.61	0.90
Cocaine	$\hat{\beta}$	−0.004	−0.009	− 0.037
	SE($\hat{\beta}$)	0.009	0.009	0.011
	*p-*value	0.64	0.35	**<.001**
Alcohol	$\hat{\beta}$	0.026	0.025	0.020
	SE($\hat{\beta}$)	0.007	0.007	0.008
	*p-*value	**<.001**	**<.001**	**0.01**
Marijuana/hashish	$\hat{\beta}$	0.006	0.005	−0.004
	SE($\hat{\beta}$)	0.009	0.009	0.010
	*p-*value	0.49	0.59	0.68
Stimulants	$\hat{\beta}$	−0.067	−0.070	−0.092
	SE($\hat{\beta}$)	0.010	0.010	0.012
	*p-*value	**<.001**	**<.001**	**<.001**
Other drugs	$\hat{\beta}$	0.049	0.049	0.048
	SE($\hat{\beta}$)	0.010	0.010	0.012
	*p-*value	**<.001**	**<.001**	**<.001**

*p* < 0.05 is presented in bold

**Fig. 2:**
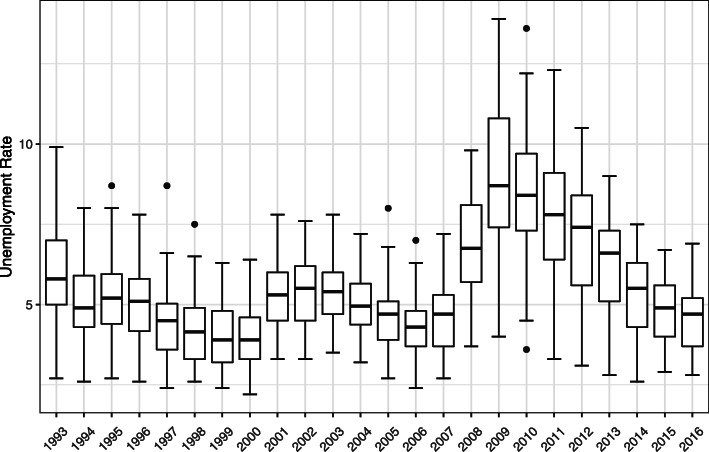
Trend of average state unemployment rate, 1993–2016

Table 3 shows the moderation analysis of how the economic recession affected the relationship between state unemployment rates and substance abuse admissions (Model 5). The unemployment rate was at a peak during the two recession periods (2001, 2008–09) (Fig. 2). Although there was a negative association between the unemployment rate and substance use admissions for stimulants ($\hat{\beta}$ = − 0.067, *p <* .01) in the baseline analysis (Model 1), the interaction term showed that the negative association was weakened during the economic recession. The moderation effect was not significant for other substances.

**Table 3:** Association between unemployment rate and substance abuse treatment admissions in economic recessions

Model 5		Unemployment rate	Economic recession	Unemployment rate* Economic recession
Opiates	$\hat{\beta}$	−0.008	0.058	0.010
	SE($\hat{\beta}$)	0.010	0.103	0.010
	*p-*value	0.43	0.57	0.67
Cocaine	$\hat{\beta}$	−0.039	0.049	0.029
	SE($\hat{\beta}$)	0.049	0.112	0.016
	*p-*value	**< 0.01**	0.66	0.07
Alcohol	$\hat{\beta}$	0.010	0.015	0.014
	SE($\hat{\beta}$)	0.008	0.082	0.012
	*p-*value	0.20	0.86	0.24
Marijuana/hashish	$\hat{\beta}$	−0.012	−0.006	0.019
	SE($\hat{\beta}$)	0.010	0.104	0.015
	*p-*value	0.22	0.96	0.20
Stimulants	$\hat{\beta}$	−0.085	−0.214	0.043
	SE($\hat{\beta}$)	0.012	0.127	0.018
	*p-*value	**< 0.01**	0.09	**0.02**
Other drugs	$\hat{\beta}$	0.041	0.178	−0.01
	SE($\hat{\beta}$)	0.012	0.125	0.020
	*p-*value	**< 0.01**	0.16	0.50

*p* < 0.05 is presented in bold

## Discussion

The present study found that the unemployment rate was significantly associated with substance abuse treatment admissions for alcohol, marijuana, opiates, cocaine, and other drugs. These results are in line with a prior study that found that as county unemployment rates increased, the opioid death rate and opioid overdose emergency department visit rate both increased [27]. Additionally, prior studies reported increases in alcohol-related disorders and problematic drinking during the recession, particularly among households experiencing unemployment [9, 28]. A recent Spanish study also found an increase in marijuana and cocaine use during the great recession [29]. Our results showed a negative association between unemployment rates and substance abuse admissions for stimulants; however, the relationship was altered during economic recessions. In prior research, being unemployed or working part-time was associated with increased use of stimulants [30]. A possible explanation for this discrepancy is the perception of stimulants compared to other drugs. For example, one study found that college students often perceive the misuse of stimulants as safe [31], and thus may not feel the need to seek treatment, especially during times of economic hardship.

Our findings lend support to the idea that the self-medication model may be applicable to examining substance use among those experiencing economic hardship. The self-medication model of drinking suggests that alcohol is used to cope with psychological distress. Economic hardship has been associated with depression, anxiety, and psychological distress [28, 32, 33]. Based on our results, this theory may also extend to illicit drug use. As a result of economic hardship, some may develop problems with alcohol and drugs due to self-medication, resulting in the increased rates of treatment admissions we found in our study. Particularly, unemployment may increase psychological distress, resulting in increasing use of and treatment admissions for marijuana, opiates, cocaine, and other drugs.

We found that the relationship between unemployment rate and annual substance abuse admissions was mostly similar (symmetric effect) for economic downturns and upturns (with the exception of marijuana/hashish, cocaine, and stimulants). These findings suggest that, at least for some substances, the unemployment rate is consistently associated with treatment admissions regardless of the current economic climate. Our findings that substance use treatment admission is associated with unemployment indicate that during economic downturns, people may rely on substances to cope. During economic crises and times of high unemployment rates, there may be a need for more substance use treatment facilities as problematic substance use increases. This study’s findings suggest the need to prioritize funding for substance use treatment facilities during and after economic crises.

The present study has some limitations worth noting. We used substance treatment facility admissions as an indicator of excessive or problematic substance abuse, which only captures a portion of substance abuse problems and may have resulted in selection bias. In addition, because of the precautions taken to ensure anonymity, some of those admitted to treatment facilities could have been double-counted if they returned for a second round of treatment. Due to the nature of the current study, caution is needed when applying grouped results to the individual level (ecological fallacy). Despite its limitations, the current study expands the existing literature work by using objective measures of problematic substance use and more diverse illicit drug categories.

## Conclusions

We found that unemployment was associated with substance abuse admissions for alcohol, marijuana, opiates, cocaine, and other drug use. These findings suggest that economic hardship is associated with increased substance use and also implies that treatment for substance use of certain drugs and alcohol should remain a priority even during economic downturns. Treatment for stimulant use may be the exception, as we found that state unemployment rates were negatively associated with treatment admissions for stimulants. However, the relationship between the unemployment rate and stimulant treatment admissions may be moderated by economic recession.

## Data Availability

Data is publicly available on https://www.icpsr.umich.edu/icpsrweb/ICPSR/series/56

## Abbreviations

TEDS: Treatment Episode Data Sets
SAMHSA: Substance Abuse and Mental Health Services Administration
ACS: American Community Survey
CPS ASEC: Current Population Survey Annual Social and Economic Supplement
NCI: National Cancer Institute
DID: Difference-in-difference
